# Sevoflurane Alleviates Myocardial Ischemia Reperfusion Injury by Inhibiting P2X7-NLRP3 Mediated Pyroptosis

**DOI:** 10.3389/fmolb.2021.768594

**Published:** 2021-10-26

**Authors:** Jiaxuan Wu, Wenfeng Cai, Ruiming Du, Haiyang Li, Bin Wang, Yanqiong Zhou, Daifei Shen, Huimin Shen, Yang Lan, Lesi Chen, Xiaoxia Zheng, Danmei Huang, Ganggang Shi

**Affiliations:** ^1^ Department of Anesthesiology, Second Affiliated Hospital of Shantou University Medical College, Shantou, China; ^2^ Department of Pharmacology, Shantou University Medical College, Shantou, China

**Keywords:** sevoflurane, myocardial ischemia reperfusion, hypoxia and reoxygenation, P2X7, NLRP3, pyroptosis

## Abstract

Myocardial ischemia is common in aging population. This study investigates the protective effect of Sevoflurane on myocardial ischemia reperfusion injury (MIRI) and its underlying mechanism. A total of 87 patients with a history of myocardial ischemia who underwent abdominal surgery with Sevoflurane general anesthesia were recruited in the study. The clinical data, blood pressure, heart rate, pressure-rate quotient (PRQ) and rate-pressure product (RPP) were recorded. Serum samples were collected and heart-type fatty acid binding protein (H-FABP), ischemia modified albumin (IMA), interleukin-1β (IL-1β), and interleukin-18 (IL-18) were measured to observe whether Sevoflurane anesthesia had protective effect on myocardium. In addition, MIRI rats and hypoxia/reoxygenation (H/R) injury cell model was established using neonatal rat ventricular myocytes (NRVM). Rats or NRVM were pretreated with sevoflurane for 45min before hypoxia. The mRNA expression of purinergic receptor-7 (P2X7) and NLR family pyrin domain containing 3(NLRP3) were examined. The protein expression of P2X7, NLRP3, apoptosis-associated speck-like protein (ASC), cysteine aspartic acid specific protease-1(Caspase-1), Gasdermin-D (GSDMD), Bcl-2 Associated X Protein (Bax), B-cell lymphoma-2 (Bcl-2) in myocardial tissue and cells were evaluated. The serum contents of IL-1β, IL-18, Malondialdehyde (MDA), Superoxide dismutase (SOD), Lactate dehydrogenase (LDH), Creatine kinase (CK), and Creatine kinase isoenzymes (CK-MB) were measured. The cellular localization and fluorescence intensity of NLRP3 and ASC in cells were detected. It was found that the secretion of IL-1β and IL-18 decreased in the patients. After I45 min/R3h in SD rats and H3h/R1h in NRVM, the protein expressions of P2X7, NLRP3, ASC, Caspase-1 and GSDMD were increased, the release of IL-1β, IL-18, CK, CK-MB, LDH and MDA were increased, and SOD activity was decreased. Sevoflurane treatment inhibited the high expression of P2X7, NLRP3, ASC, Caspase-1 and GSDMD, inhibited the release of LDH, CK,CK-MB and MDA in cells, and improved the activity of SOD, indicating that Sevoflurane alleviated the damage of MIRI of rats and H/R of NRVM, and had myocardial protective effect. Taken together, our study suggests that Sevoflurane inhibited the expression of IL-1β, IL-18 and GSDMD by inhibiting the P2X7-NLRP3 signaling pathway. It reduced the H/R injury of cardiomyocytes and protected the cardiac function by regulating inflammatory reaction and pyroptosis.

## Introduction

Sevoflurane is an inhalation anesthetic drug. It induces anesthesia with quick wake up and has little effect on circulatory and respiratory inhibition. It was reported that Sevoflurane has protective effect on myocardial ischemia reperfusion injury (MIRI) by inhibiting inflammatory reaction (Nader et al., 2004; Symons and Myles, 2006; Yao et al., 2010). The function of Sevoflurane may be due to its effect on the lipid solubility of cell membrane, leading to membrane expansion. Sevoflurane reduces the expression of Cluster of Differentiation 11b (CD11b) on the surface of neutrophils and inhibits the adhesion of neutrophils to endothelial cells (Li et al., 2020). It also reduces the number and activity of neutrophils captured during cardiac reperfusion, reduces the adhesion between platelets and vascular endothelia after ischemia, and reduces the secretion of pro-inflammatory cytokines Tumor necrosis factor-alpha (TNF-α), IL-1β and others.

The ischemic myocardium may aggravate the structural damage of the heart after reperfusion, causing cell death. This might further damage the cardiac function and affect the prognosis of patients. After myocardial cell injury, mitochondrial function is impaired, and adenosine triphosphate (ATP) is released from the damaged or dead cell to act on the P2X7 receptor on cell membranes (Peng et al., 2015; Shokoples et al., 2021). An important characteristic of P2X7 is that the non-selective ion channels will open under the action of high extracellular ATP concentration, allowing macromolecules to pass through, and leading to the formation of NLRP3 inflammasome (Yin et al., 2017; Zhou et al., 2021) NLRP3 is closely associated with MIRI (Hu et al., 2019). NLRP3 can promote the release of inflammatory mediators from cardiomyocytes, such as IL-1β and IL-18, and induce pyroptosis (Ji et al., 2021). Pyroptosis is frequently involved in the development of atherosclerotic heart disease, infectious diseases and nervous system-related diseases, that are characterized by the formation of inflammasome, the activation of gasdermin and caspase-1, and the release of pro-inflammatory factors.

During the first 6 h of reperfusion after acute myocardial infarction, substances released from the infarct area can recruit neutrophils to the infarct area (Liu et al., 2016; Sorriento and Iaccarino, 2019). Within 24 h, the neutrophils migrate to the myocardium, causing inflammatory response, blockage of blood vessels and release of various degrading enzymes, leading to myocardial damage. Treatment of the myocardium with blood containing white blood cells or anti-ICM1 antibodies during reperfusion reduced the extent of myocardial infarction. Inhibition of inflammatory response plays an important role in alleviating MIRI. The activation of neutrophils increased significantly during reperfusion, but decreased significantly after Sevoflurane pretreatment. Sevoflurane pretreatment can improve the myocardial contraction force, reduce arrhythmia, improve left ventricular pressure, decrease left ventricular end-diastolic pressure at the same time, and by reducing coronary artery (including collateral circulation) resistance to improve myocardial perfusion and reduce the area of myocardial infarction (De Hert et al., 2004; Garcia et al., 2005; Lurati Buse et al., 2012).

In this study, clinical data and serum samples were collected from patients with either ST-T abnormality in preoperative ECG, or have symptoms or history of myocardial ischemia, who underwent abdominal surgery with general anesthesia to observe whether Sevoflurane has a cardioprotective effect in these patients. In addition, the primary cardiomyocyte H/R model and the rat MIRI model were established to observe the effect of Sevoflurane on the expression of P2X7 and NLRP3, to investigate how Sevoflurane inhibited the release of IL-1β and IL-18 and reduced pyroptosis through P2X7-NLRP3, and to explore the mechanism of Sevoflurane protects against MIRI in cardiomyocytes.

## Materials and Methods

### Chemicals and Reagents

Midazolam (Batch No.MZ210112, Jiangsu NHWA Pharmaceutical Co.,Ltd, China), Sufentanil (Batch No.11A01111, Yichang Renfu Pharmaceutical Co., Ltd. China) and Remifentanil (Batch No.00A12301, Yichang Renfu Pharmaceutical Co., Ltd. China), Propofol (Batch No.10PK7241, Guangdong Jiabo Pharmaceutical Co., Ltd. China), Rocuronium bromide (Batch No.2010041.1, Zhejiang Xianju Pharmaceutical Co., Ltd. China), Sevoflurane (Batch No.20122331, Jiangsu Hengrui Pharmaceutical Co., Ltd. China),PrimeScripttr RT reagent Kit with gDNA Eraser (Lot# AK3801,Takara, Japan) and SYBR Premix Ex TaqTM Ⅱ(Lot# AKA303,Takara, Japan), P2X7 Rabbit Polyclonal Antibody (Lot# GR3276506Abcam, UK)and NLRP3 Rabbit Polyclonal Antibody (Abcam, UK); Caspase-1 mouse monoclonal Antibody (Lot# J2919,Santa Cruz Biotechnology,United States),ASC mouse monoclonal Antibody (Lot# K1920,Santa Cruz Biotechnology,United States), GSDMD mouse monoclonal Antibody (Lot# GR3281507-4,Santa Cruz Biotechnology,United States), GADPH Rabbit Monoclonal Antibody (Lot# 201050119, Beijing ZSGB- Biotechnology Co., Ltd.Chian), HRP-labeled sheep anti-mouse IgG (Lot# A0521,Wuhan  BOSTER Biological Technology co.ltd. China),IL-1β ELISA Kit (Lot# 226593-003,Wuhan Boster Biological Technology. Ltd, Wuhan, China) and IL-18 ELISA Kit (Lot# 2002-6631,Wuhan Boster Biological Technology. Ltd, Wuhan, China), LDH (Lot# 20200706, Nanjing Jiancheng Institute of Biological Engineering, China), MDA (Lot# 20210130, Nanjing Jiancheng Institute of Biological Engineering, China)and SOD kit (Lot# 20200511,Nanjing Jiancheng Institute of Biological Engineering, China),2,3, 5-Triphenyl Tetrazolium Chloride and Evans Blue (Lot# BCCD5244,Sigma-Aldrich, St. Louis, Mo, United States).

### Study Subjects

This study was approved by the Ethics Committee of Shantou University Medical College. A total of 87 patients with a history of coronary heart disease (diagnosed based on the Diagnostic criteria from the 2014 ACC/AHA/AATS/PCNA/SCAI/STS stability of ischemic heart disease diagnosis and treatment guidelines and ASA Class II-III) and underwent abdominal surgery were included in the study. All patients signed the informed consents. Patients aged 18–65 years old. They were randomly divided into two groups according to the random number table: Sevoflurane group (n = 56) and Propofol group (n = 31). Another 30 individuals with no history of myocardial ischemia who underwent abdominal surgery with Sevoflurane anesthesia were used as the control group.

### Anesthesia Method

Clinical evaluation and medical record review were conducted before surgery. The basic information and past medical history of patients were recorded. All patients completed routine preoperative cardiac examination, including a 12-lead electrocardiogram, chest X-ray, echocardiography, and laboratory examinations.

After the patients were admitted to the operating room, they were monitored with electrocardiogram, systolic blood pressure (SBP), diastolic blood pressure (DBP), SPO_2_, and PetCO_2_. Anesthesia induction for all patients was performed using midazolam at the dose of 0.03–0.05 mg/kg, sufentanil 0.1–0.2 μg/kg, propofol 1.5–2.0 mg/kg and rocuronium 0.6–0.9 mg/kg. Volume control ventilation was performed after endotracheal intubation. The tidal volume was 6–8 ml/kg, the respiration ratio was 1:2, and the oxygen flow was 1.5 L/min. The ventilation frequency was adjusted to maintain the PetCO_2_ at 35–45 mmHg. Sevoflurane group was treated with inhalation Sevoflurane 1.0–1.5 minimum alveolar concentration (MAC) for anesthesia maintenance. The Propofol group was maintained with continuous intravenous infusion of propofol of 50–200 μg/kg/min. Intraoperative intravenous infusion of remifentanil 0.05–0.20 μg/kg/min was used to maintain analgesia.10∼20 mg rocuronium was added in a single dose as needed.

### Observation Indicators

Blood pressure, heart rate, SPO_2_, and PetCO_2_ were continuously monitored. SBP, DBP and HR were recorded at the time when patients entering the operating room, at the beginning of anesthesia, and every 5 min during anesthesia until the end of anesthesia. RPP and PRQ were calculated. Electrocardiogram examination was performed to analyze the changes of ST-T in electrocardiogram at the end of anesthesia, 24 h Post-anesthesia and 72 h Post-anesthesia, and the changes of ST-T segment in 12-lead electrocardiogram were more pronounced (ST-segment depression or elevation, T-wave inversion or biphasic) than those in baseline electrocardiogram Pre-anesthesia.The use of Sevoflurane and the recovery quality were recorded. Blood samples were collected Pre-anesthesia and Post-anesthesia, 24 and 72 h Post-anesthesia. Serum was separated and IMA, H-FABP, IL-1β and IL-18 were measured. The incidence of unstable angina pectoris, myocardial infarction, heart failure and cardiogenic death were recorded after 30 days.

### Animals and Myocardial Ischemia-Reperfusion Model

Animal experiments were approved by the Animal Ethics Committee of Shantou University Medical College. Male Sprague Dawley (SD) rats weighing 250–300 g were obtained from the Laboratory Animal Center of Shantou University Medical College. All animals had free access to food and water, and the room temperature was maintained between 23°C and 24°C. Sevoflurane inhalation or intraperitoneal injection of 1% Pentobarbital Sodium were used to minimize animal suffering during the procedure.

Eight-week-old male SD rats, weighed about 250–300 g, were used. After the rats were completely anesthetized by intraperitoneal injection of 1% Pentobarbital Sodium, the chest hair was removed, the rats were fixed on the experimental table and connected to the lead ECG. The rats were inserted with the endotracheal tube and the respiratory parameters were adjusted (ALC-V8S, Shanghai Alcott Biotechnology Co., Shanghai, China). After the chest was disinfected with alcohol, a longitudinal incision was made between the third and 4^th^ intercostals on the left. Blood vessels were visible at 1–2 mm from the lower edge of the left atrial appendage. A bead with a diameter of 0.3 cm was threaded on the 5-0 silk suture, and the left anterior descending (LAD) was ligated with the silk suture. The silk suture was ligated so that it compresses LAD for 45 min. ST segment elevation was more than 2/3 and T wave was high in ECG during ischemia. Myocardium at ligation site was white. Myocardial perfusion can be restored by cutting the suture and removing the beads. The elevated ST segment on the ECG decreased by more than 1/2, and the T wave gradually recovered, indicating successful reperfusion.

### Animal Experiment Grouping

**FIGURE 1:**
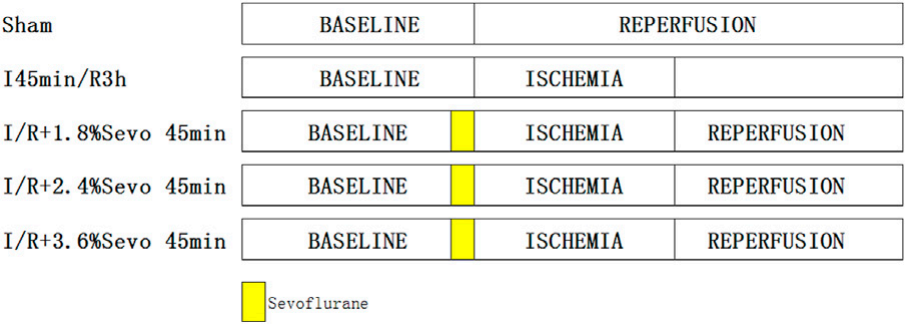
Rats in each group.In the sham group, the silk suture was passed under LAD without ligation. The other groups were ligated with LAD for 45 min and then reperfused for 180 min. Sevoflurane groups with different concentration of sevoflurane was inhaled with corresponding concentrations for 45 min before ischemia.

SD rats were randomly divided into the following five groups (Figure 1): sham group, ischemia/reperfusion (I/R) group, and I/R + sevoflurane groups. In the sham group, the silk suture was passed under LAD without ligation. The other groups were ligated with LAD for 45 min and then reperfused for 180 min. The 1.8% sevoflurane, 2.4%sevoflurane, 3.6%sevoflurane group was inhaled 1.8% sevoflurane (0.7 MAC), 2.4% sevoflurane (1.0 MAC) and 3.6% sevoflurane (1.3 MAC) respectively for 45 min before ischemia.

### ECG Recording and Cardiac Ultrasound

Animal eight-channel physiological recording system (China Ede Company, PL3508/P 75dB/16 bits) was used to collect electrocardiogram, ventricular pressure and arterial pressure. The ST segment changes in ECG and the occurrence of arrhythmias during ischemia/reperfusion was recorded.

The cardiac function of rats was detected by small animal multimode imaging system (Fuji, Vevo^®^ LAZA2100, Japan). The images of long axis and short axis of the longitudinal section of the hearts of rats were collected by LZ250 ultrasound probe. The images were analyzed to obtain the values of left ventricular ejection fraction (LVEF) and left ventricular fraction shorting (LVFS). These results reflect the changes of heart function of rats in each group after different treatments.

### Measurement of Myocardial Infarct Size

The left anterior descending branch of the heart of rats was ligated, and 1% Evans blue solution was injected into the right atrium. After the limbs were blue stained, the hearts were cut off and frozen at −30°C for 1 h. The hearts were cut into sections about 2 mm and incubated in 1.5% 2,3,5-triphenyltetrazolium chloride (TTC) solution at 37°C for 20 min and fixed with 4% paraformaldehyde for 4 h. The normal perfusion area was stained blue by Evans blue, and the myocardium in the ischemic area was stained red by TTC. The infarct area is white because the cell membrane of cardiomyocytes is damaged, and the release of intracellular dehydrogenase cannot be stained red by TTC. After taking photos, the staining results were analyzed with Image Pro Plus 6.0 software, and the percentage of infarct myocardium in ventricular area was calculated.

### Isolation of Primary Cardiomyocytes NRVM and Modeling of Hypoxia/Reoxygenation NRVM

SD rats 1–5 days old were anesthetized, their chest cavities were opened, hearts were removed, bloods were washed with PBS, and the hearts were cut into tissue blocks of 1–3 mm. After digestion with trypsin and type II collagenase, the heart cells were collected. The fibroblasts were removed and 5-bromodeoxyuridine was added into the medium to inhibit the growth of fibroblasts. After that, purified cardiomyocytes were obtained.

After 3–4 days of culture, NRVM were washed 3 times with PBS. 100% nitrogen was added into the anoxic solution and the anoxic solution was saturated for 20–30 min. After saturation, the original culture medium in the Petri dish was removed and cleaned with anoxic solution for 2–3 times. Different volumes of anoxic solution were added according to the size of the Petri dish. The cells were placed in a hypoxic/anaerobic workstation for 3 h under the condition of 1% O_2_, 5%CO_2_ and 94% N_2_ at 37°C to establish the anoxic cell model. After 3 h of anoxia, the anoxic fluid was removed, and the oxygenated fluid was used to clean the cells 2–3 times, and the oxygenated fluid was replaced in the cell culture dish. The reoxygenation model was completed after being incubated for 1 h in an incubator at 37°C and 5% CO_2_.

### Real-Time Quantitative PCR

Real-time quantitative PCR was used to detect the mRNA expression of P2X7 and NLRP3 in myocardial cells. Total RNA of cardiomyocytes was extracted according to the instructions of RNAiso Plus and reversely transcribed into cDNA. The specific primers of rat P2X7 and NLRP3 were used for real-time quantitative PCR (Table 1).

**TABLE 1:** Primer sequences.

Primer	Primer sequences (5′-3′)
P2X7	forward:AGTCTGCAAGATGTCAAAGG
	reverse: ATT​TCC​TCA​GGT​TGT​CCA​G
NLRP3	forward: TTC​CCA​GAC​CCT​CAT​GTT​GC
	reverse: CAG​GGC​ATT​GTC​ACT​GAG​GT
GAPDH	forward: TCA​AGA​AGG​TGG​TGA​AGC​AG
	reverse: AGG​TGG​AAG​AAT​GGG​AGT​TG

### Western Blot Analysis

The cells were lysed with RIPA solution, and the total cell proteins in each group were extracted. The BCA protein detection kit was used to measure the protein concentration of each sample to ensure that the loading total protein in each group was the same. After SDS-PAGE separation, the proteins were transferred onto the nitrocellulose (NC) filter membrane. After the electric transfer, the membrane was sealed with 5% skim milk for 1 h. After sealing, membrane was treated with P2X7 (1:300), NLRP3 (1:300), ASC (1:100), Caspase-1 (1:100), and GSDMD1 (1:3,000) antibodies and incubated overnight at 4°C. Then the membranes were washed with TBST for 3 times, 10 min each time. The goat anti-rabbit-IgG-HRP antibody (1:20,000) and goat anti-murine-IgG-HRP antibody (1:20,000) diluted in 5% skim milk were added, and incubated at room temperature for 1 h. The membranes were washed again with TBST for 3 times, 10 min each time. Finally, chemiluminescent solution was used for exposure in a dark room. Gel-Pro image analysis software (Media Cybernetics, United States) was used to analyze the optical density of the protein bands.

### ELISA Detected Blood Cytokines

Blood samples were collected from the patients using a collector vessel with separation gel. Serum was obtained by centrifugation at 3,000°r/min for 5°min, and stored at −80°C. Cardiac injury indexes such as CK, CK-MB, LDH, IMA and H-FABP in serum at different time points were determined according to the instructions of the ELISA detection kits. In addition, the levels of IL-1β and IL-18 were detected. At the end of the experiment, the abdominal cavity was opened and blood was collected from the inferior vena cava. Serum was obtained by centrifugation at 3,000°r/min for 5 min and stored at −80°C. For the cell experiment, cell culture medium was collected from each group and centrifuged at 2000 r/min for 5 min to remove cell debris. The levels of inflammatory cytokines IL-1β and IL-18 in serum or cell culture medium were measured according to the instructions of ELISA detection kits.

### Measurement of MDA, SOD and LDH Level

MDA content in cardiomyocytes was detected by thiobarbituric acid assay. The activity of SOD in myocardial cells was detected by xanthine oxidase. The content of MDA and SOD reflects the ability of scavenging oxygen free radicals. The content of lactate dehydrogenase (LDH) in the supernatant of cultured cells was determined by colorimetric method to reflect the extent of damage to cardiomyocytes.

### Immunohistochemical and Immunofluorescent Staining

After 3 h of reperfusion, the hearts of rats were taken by thoracotomy. The myocardial tissue below the ligation line was cut, fixed in 10% formaldehyde, dehydrated, embedded in paraffin, and sectioned at the thickness of 4 μm. Paraffin sections were placed in the oven at 65°C for 30 min, and then dewaxed with xylene and dehydrated with alcohol. After heat antigen repair, the endogenous enzyme activity was inactivated with 3% H_2_O_2_ solution, block with goat serum for 1 h. The primary antibody anti-NLRP3 (1:100) or anti-GSDMD (1:1,000) were added and incubated overnight at 4°C. The HRP labeled second antibody was added, incubated at 37°C for 30 min, and proceed for DAB color development. Sections were counterstained with hematoxylin, and sealed with neutral gum. Sections were observed under the microscope. Five visual fields were randomly selected from each section under the same conditions, Image Pro Plus 6.0 was used for image analysis, and the number of positive cells was calculated.

The colocalization of NLRP3 and ASC protein was detected by immunofluorescence. NRVM were inoculated on the coverslip in a 24-well plate and cultured for 72 h. After that, the cells were fixed with 4% paraformaldehyde for 15 min, washed with PBS for 3 times, and incubated with TritonX-100 for 10 min. After the normal goat serum was sealed for 1 h, the diluted NLRP3 (1:100) and ASC (1:100) antibodies were added and placed at 4°C overnight. The samples were taken out the next day, soaked in 0.1% Tween20 containing PBS for 3 times, and incubated with the secondary antibodies Alexa Fluor 488 (1:200) and Alexa Fluor 594 (1:300) for 1 h. Then, sections were washed for three times, and Hoechst33342 was used to stain the nucleus for 15 min. Finally, the slides were cover-slipped. The immunofluorescence intensity was detected by confocal microscope, and the images were analyzed by Image Pro Plus 6.0 software.

### ROS Assay Assessment

The OCT in frozen sections of rat hearts was washed off with PBS. The Dihydroethidium (DHE) dye was prepared by diluting the original DHE solution with PBS solution. The diluted DHE dye (5 μM) 50 μL was added to each slide and incubated for half an hour in the dark. After incubation, slides were washed with PBS for 3 times to stop dyeing. The glass slides were dried at room temperature, and anti-fluorescence quenching solution was added to the slides and covered with coverslip. The staining results were observed under a microscope. The number of DHE-positive nuclei were measured in three random visual fields.

### TUNEL Assay

NRVM were cultured in coverslips for 4–5 days, and then grouped according to the experimental requirements. The coverslip was fixed in 4% formaldehyde solution for 15 min, washed with PBS for 3 times, 5 min each. Then, 100 μL of 20 μg/ml protease K solution was added, incubated at room temperature for 5 min, and washed with PBS for 10 min. The excess liquid was removed from the section and 100 μL equilibrium buffer was added to cover the heart tissue, and balanced at room temperature for 10 min in the dark. The 50 μL rTDT incubation buffer (equilibrium buffer: nucleoside mixture: rTDT = 45:5:1) was added and incubated in an incubator at 37°C for 1 h. After incubation, the coverslip was immersed in 2×SSC and incubated at room temperature for 15 min to terminate the reaction. Slides were washed with PBS for 3 times, 5 min each. The anti-fluorescence quenching solution containing Hoechst33342 was added. The apoptotic cells were observed with green fluorescent filter under a fluorescence microscope. The number of apoptotic cells and the total number of cells were counted and the apoptotic rate was calculated.

### Statistical Analysis

The data were statistically analyzed using the Graphpad prism 6.0 (San Diego, California, United States). The measurement data were expressed as mean ± SD. The data at different time points in the same group or between different groups were statistically analyzed by one-way analysis of variance (ANOVA), then the data between multiple groups were compared by Tukey’s test. When *p* < 0.05, the difference was considered as of statistical significance.

## Results

### Sevoflurane Alleviates Myocardial Injury in Patients With Myocardial Ischemia

#### Demographic Characteristics

The study included 87 patients with a history of myocardial ischemia and 30 cases of individuals with no history of myocardial ischemia. In the 30 cases, there were 13 males and 17 females, aged 18–65 years old. In the 87 cases, Sevoflurane group consisted of 56 cases, 29 males and 27 females, and Propofol group consisted of 31 cases, 17 males and 14 females, aged 18–65 years old. There was no significant difference in gender and age among these three groups (*p* > 0.05). The demographics of participants by group (Control, Sevoflurane vs Propofol) including age, gender, NYHA Cardiac function classification, hypertension, diabetes, myocardial infarction and smoking were summarized (Table 2).

**TABLE 2:** Demographic characteristics of Groups (%).

	Control	Sevoflurane	Propofol
Number	30	56	31
Age (year)	56.8 ± 14.2	53.2 ± 15.8	51.2 ± 13.6
$\bar{\text{x}}$ ± s			
M:F	13:17	29:27	17:14
NYHA Cardiac function classification ≥ III	5 (16.7)	11 (19.6)	7 (22.6)
History of HTN	6 (20)	16 (28.6)	10 (32.2)
History of DM	6 (20)	16 (28.6)	8 (25.8)
History of MI	0 (0)	5 (8.9)	2 (6.5)
History of Smoking	11 (36.7)	19 (33.9)	11 (35.5)

MF, Male:femal8; NYHA, New York Heart Association; MI, myocardial infarction; HTN, hypertension; DM, diabetes mellitus.

### Changes of Vital Signs in These Three Groups at Different Time Points peri-Anesthesia

**FIGURE 2:**
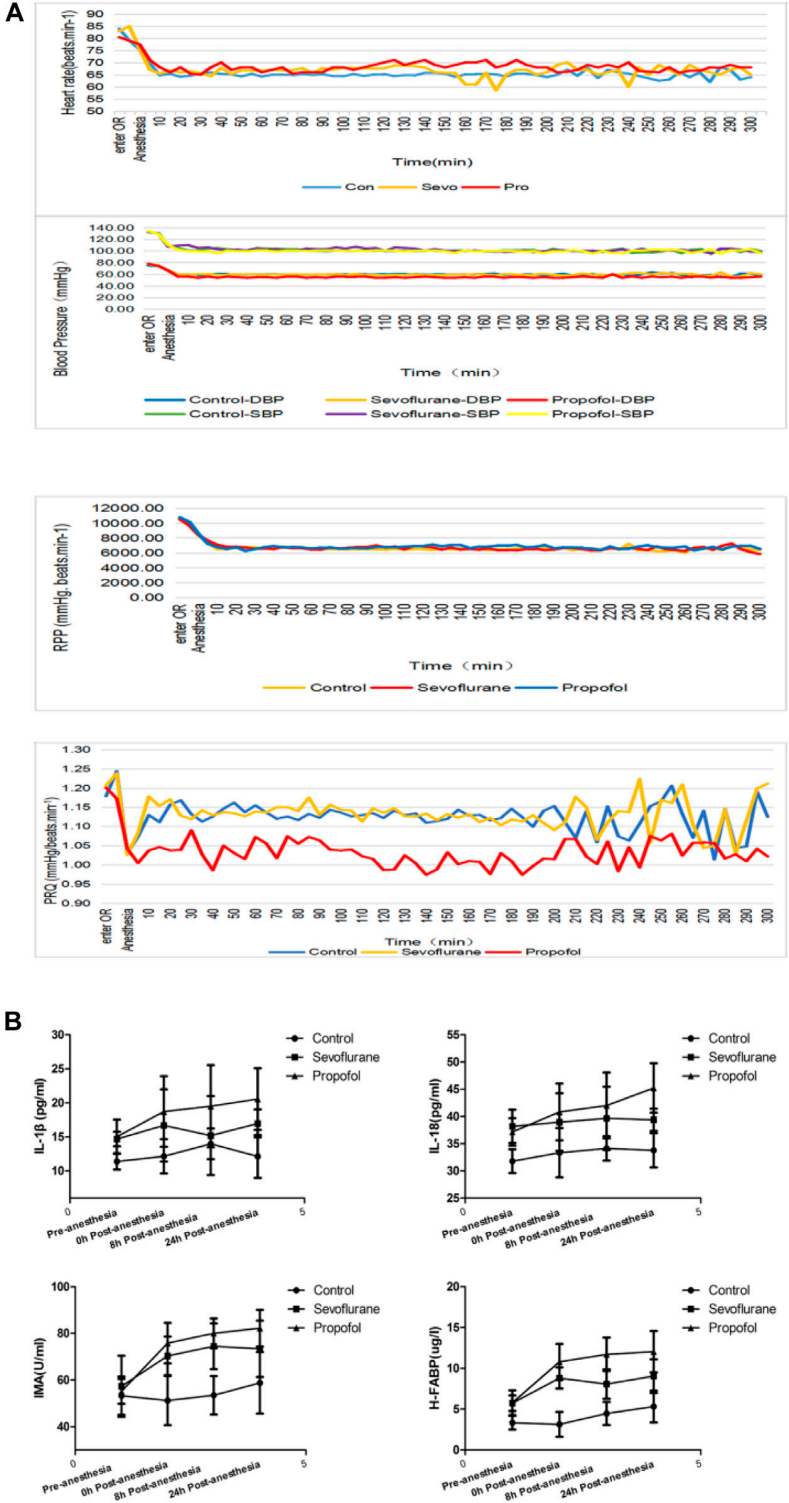
Changes in vital signs and inflammatory factors in these three groups of patients **(A)** different general anesthetics on the changes of heart rate, blood pressure, RPP and PRQ in peri-anesthesia in the three groups **(B)** Changes of IL-18 at different time points at pre- and post-anesthesia.

The BP and HR of patients in these three groups decreased after anesthetic induction due to the effects of sevoflurane and propofol on BP and HR. At each time point in anesthesia, BP and HR were lower than that before anesthesia. Propofol had more prominent effect in inhibiting blood pressure (Figure 2A). RPP>12,000 and PRQ<1.0 indirectly reflected myocardial oxygen consumption during the operation. Calculation RPP and PRQ showed that RPP<12,000 and PRQ>1.0 could be achieved in all the three groups, but PRQ was close to one in the propofol group (Figure 2A), which might be related to the effect of propofol on blood pressure inhibition.

### Changes of Myocardial Injury and Inflammatory Factors in These Three Groups During Peri-anesthetic Periods

Both IMA and H-FABP are early diagnostic markers of myocardial damage following myocardial ischemia. Compared with the control group, IMA and H-FABP increased in sevoflurane and propofol groups at 0, 8 and 24 h post-anesthesia, with statistical significance (*p* < 0.05). The increase was more obvious in the propofol group, with statistical significance (*p* < 0.05) (Figure 2B). The levels of IL-1β and IL-18 in serum were determined by ELISA. Compared with the control group, the levels of IL-1β and IL-18 in patients in the sevoflurane and propofol groups were increased at 8 and 24 h post-anesthesia, with statistical significance (*p* < 0.05). Compared with the sevoflurane group, the increase was more obvious in the propofol group, with statistical significance (*p* < 0.05) (Figure 2B).

### The Incidence of Post-anesthesia Cardiovascular Events in These Three Groups

Compared with the control group, the incidence of new ST-T abnormality of ECG is higher in sevoflurane and propofol groups at immediate post-anesthesia,1^st^ day and 2^nd^ day post-anesthesia. This might be related to the patient’s history of myocardial ischemia, and the myocardial function is affected by surgery and anesthesia. Compared with the propofol group, the incidence of new ST-T abnormality of ECG decreased in the sevoflurane group at the 1^st^ day and 2^nd^ day post-anesthesia, with statistical significance (P < 0.05), indicating that sevoflurane has a milder effect on cardiac function. Compared with the control group, the incidence of unstable angina was higher in the propofol group at the 30^th^ day post-anesthesia, with statistical significance (P < 0.05). Compared with the control group, the incidence of unstable angina was slightly higher in the sevoflurane group at the 30^th^ day post-anesthesia, but with no statistical significance (P > 0.05). Compared with the propofol group, the incidence of unstable angina pectoris was lower in the sevoflurane group at the 30^th^ day post-anesthesia, with statistical significance (P < 0.05). There was no significant difference in the incidence of myocardial infarction, heart failure and cardiogenic death 30 days after anesthesia among the three groups (Table 3).

**TABLE 3:** Post-anesthetic myocardial ischemia and cardiac events in the three groups (%).

Group	Subject (number)	New ST-T abnormality of ECG immediately post-anesthesia	New ST-T abnormality of ECG 1st day post-anesthesia	New ST-T abnormality of ECG 2nd day post-anesthesia	30th day post-anesthesia unstable angina	30th day post-anesthesia MI	30th day post-anesthesia CHF	30th day post-anesthesia cardiac death
Control	30	1 (3.3)	1 (3.3)	0 (0)	0 (0)	0 (0)	0 (0)	0 (0)
Sevoflurane	56	8 (14.3)^*^	10 (17.9)^*#^	10 (17.9)^*#^	2 (3.6)^#^	0 (0)	1 (1.8)	0 (0)
Propofol	31	5 (16.1)^*^	13 (35.3)^*^	13 (35.3)^*^	5 (16.1)^*^	0 (0)	1 (3.2)	0 (0)

ML, myocardial infarction; CHF, congestive heart failure; *p < 0.05 compared with Control group.#p < 0.05 compared with Propofol group.

## Sevoflurane Alleviates MIRI in Rats

### Electrocardiogram Changes in Rat MIRI Model

**FIGURE 3:**
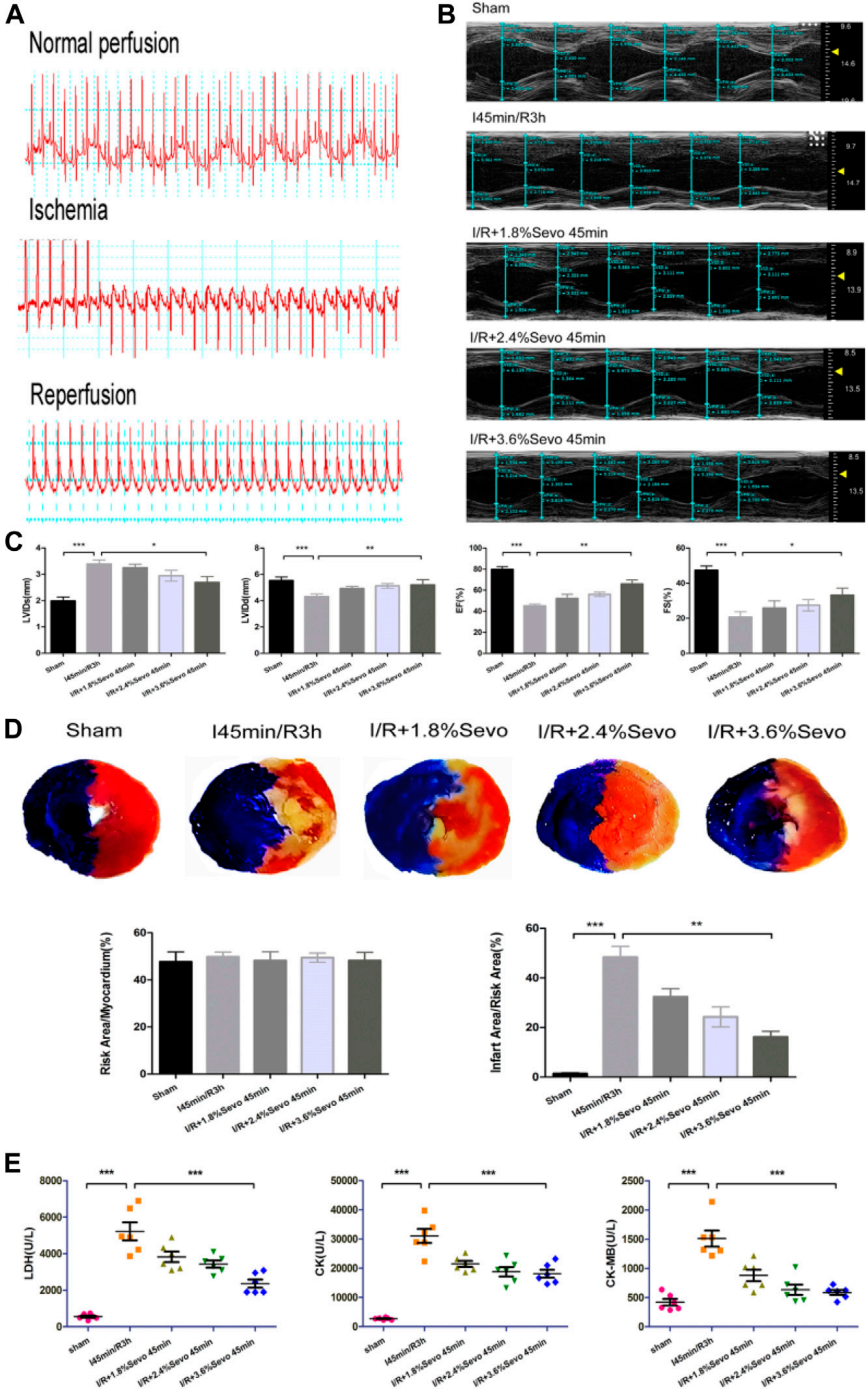
Sevoflurane alleviates MIRI in rats **(A)** Typical electrocardiogram of normal perfusion, ligation of LAD and reperfusion **(B)** Cardiac ultrasonography in rats of normal perfusion, MIRI and treatment with different concentrations of sevoflurane **(C)** Histogram of rat cardiac ultrasonography **(D)** Myocardial infarction area and histogram of rats treated with different concentrations of sevoflurane **(E)** Effects of Sevoflurane at different concentrations on the release of LDH, CK and CK-MB in myocardial tissue after MIRI. Data are expressed relative to the mean value of sham group and were presented as mean ± SD (n = 6). *p < 0.05, **p < 0.01, ***p < 0.001 vs respective controls.

After the ligation of LAD, the ECG showed typical signs of myocardial ischemia: high T wave, increased amplitude, elevated ST segment, and slow heart rate. After loosening the ligation line and reperfusion of the myocardium, the T wave on the ECG decreased rapidly, the elevated ST decreased by 30–50%, and the heart rate recovered (Figure 3A)

### Myocardial Infarction and Echocardiography

The results of cardiac ultrasound showed that compared with the Sham group, the LVEF and LVFS of the rats in the I/R group were significantly decreased, and the cardiac function was seriously damaged. However, compared with the I/R group, Sevoflurane pretreated rats showed a lower degree of LVEF and LVFS reduction after MIRI (Figure 3B, C). The results indicated that Sevoflurane pretreatment could protect the heart from injury after MIRI.

After myocardial ischemia reperfusion in rats, the area of myocardial infarction reflects the severity of myocardial ischemia and infarction after ischemia, and is used in the study of MIRI. Ischemia can cause infarction in a certain area. The size of the area depends on the duration and severity of ischemia. If reperfusion is not performed, the infarct can extend from the endocardial to the epicardial area. Although reperfusion can save some of the myocardial tissue from infarction in the risk area of ischemia, it can result in cell death and changes in myocardial infarct size (Gallagher, 1997). The size of myocardial infarction in the Sevoflurane preconditioning group was smaller than that in the I/R group and correlated with the concentration of sevoflurane. Of the three concentration groups, the 3.6% Sevoflurane group had the most significant reduction in MI area (Figure 3D).

### Contents of LDH, CK and CK-MB in Serum of Rats

After myocardial ischemia, myocardial cells are injured. Enzymes in the cells were released out of the cells due to the rupture of the cell membrane. By measuring the enzyme content in serum or medium, the degree of myocardial cell injury can be evaluated. CK-MB is the main enzyme released into the blood when cardiomyocytes are damaged. At the same time, intracellular LDH and CK are also released to the outside of the cells. The contents of LDH, CK and CK-MB in serum directly reflect the severity of cardiac function injury in rats. Compared with the Sham group, the release of myocardial enzymes was significantly increased in the I/R group and the cardiac function was seriously damaged. However, the release of myocardial enzymes in Sevoflurane group was less than that in I/R group (Figure 3E). This indicates that Sevoflurane treatment can reduce the release of myocardial enzymes after MIRI.

### Sevoflurane Alleviates Inflammatory Cell Infiltration in MIRI Rats

#### Immunofluorescence Staining of CD11b

**FIGURE 4:**
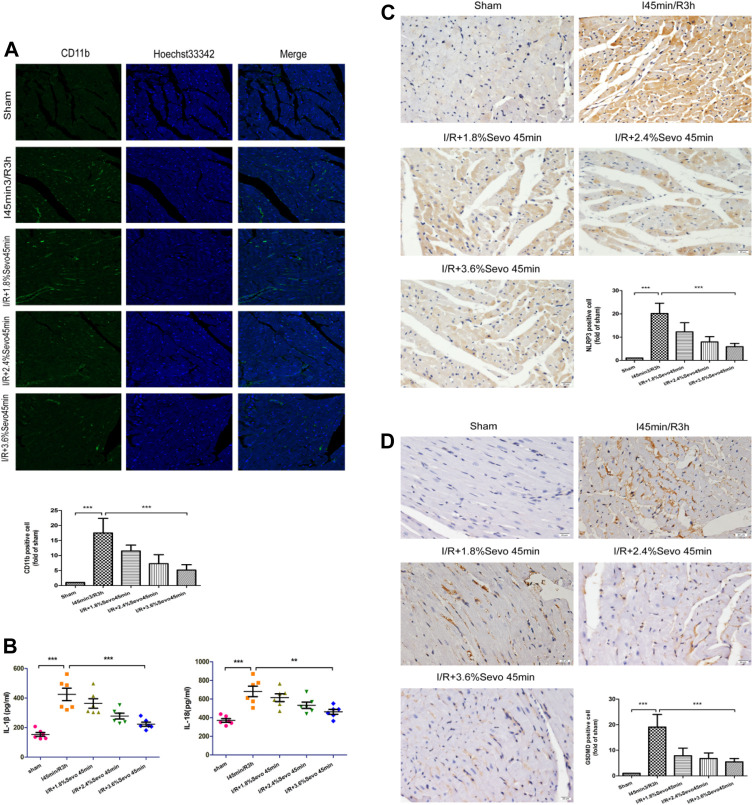
Sevoflurane alleviates inflammatory cell infiltration in MIRI rats **(A)** The expression of CD11b in rat myocardial tissue determined by immunofluorescence staining (n = 6,Scale bars 20 um) **(B)** Effects of Sevoflurane at different concentrations on the release of IL-1β and IL-18 in myocardial tissue after MIRI. All values are expressed as means ± SD (n = 6). *p < 0.05, **p < 0.01, ***p < 0.001 vs respective controls **(C)** Expression of NLRP3 in rat myocardial tissue by immunohistochemical staining of normal perfusion, MIRI and sevoflurane treatment with different concentrations in rats (n = 6,Scale bars 20 um) **(D)** The Expression of GSDMD in rat myocardial tissue by immunohistochemical staining of normal perfusion, MIRI and sevoflurane treatment with different concentrations in rats (n = 6,Scale bars 20 um).Data are expressed relative to the mean value for sham group and were presented as mean ± SD. *p < 0.05, **p < 0.01, ***p < 0.001 vs respective controls.

CD11b is the biomarker of monocytes, macrophages, neutrophils and NK cells. Positive immunofluorescent staining of CD11b showed these cells. As shown in Figure 4A, the number of CD11b-labeled inflammatory cells increased in the myocardium of rats in the ischemia-reperfusion group. However, the accumulation of inflammatory cells in Sevoflurane pretreated rats was significantly reduced compared with the rats in I/R group, with less inflammatory cell aggregation and fewer CD11b-labeled inflammatory cells.

### Serum IL-1β and IL-18 Levels in Rats

Activation of inflammasome hydrolyzes pro-caspase-1 (45 kD) into two fragments (20 kD and 10 kD) to produce active caspase-1. Pro-IL-1β and Pro-IL-18 were further cleaved by caspase-1 to generate active IL-1β and IL-18. IL-1β and IL-18 are released extracellularly, causing an amplification of inflammatory signals, and ultimately an intensification of the inflammatory response. The occurrence of this reaction can lead to the adhesion of neutrophils and monocytes and the generation of oxygen free radicals, and induce cell edema and death. Compared with the Sham group, serum IL-1β and IL-18 levels were increased in the I/R group, indicating that the production and release of inflammatory factors were increased after ischemia/reperfusion, while the values were relatively low after Sevoflurane pretreatment (Figure 4B and Table 4).

**TABLE 4:** Effects of Sevoflurane at different concentrations on the release of IL-1β and IL-18 in myocardial tissue after MIRI (n = 6).

	IL-1β(pg/ml)	IL-18 (pg/ml)
Sham	170.44 ± 13.32^#^	370.16 ± 23.36^#^
I45min/R3h	424.76 ± 54.18^*^	681.92 ± 86.36^*^
I/R+1.8%Sevo 45 min	379.78 ± 40.29^*,#^	615.16 ± 54.02^*,#^
I/R+2.4%Sevo 45 min	294.53 ± 26.03^*,#^	533.09 ± 45.88^*,#^
I/R+3.6%Sevo 45 min	256.43 ± 20.56^*,#^	462.49 ± 28.53^*,#^

*p < 0.05 vs Sham group; #*p* < 0.05 vs 145 min/R3h group.

### Immunohistochemical Staining of Myocardial Tissue

The expression of NLRP3 and GSDMD was detected by immunohistochemical staining. Compared with the Sham group, the expression of NLRP3 and GSDMD was increased in the tissue sections of I/R group. Compared with the I/R group, the expression level of NLRP3 and GSDMD in the Sevoflurane treatment group was significantly down-regulated, and the degree of reduction was related to the concentration, and the decrease was most obvious in the 3.6% Sevoflurane group (Figures 4C, D).

## Sevoflurane Alleviates Oxidative Stress and Pyroptosis in MIRI Rats

### Contents of SOD and MDA in Myocardial Tissue

**FIGURE 5:**
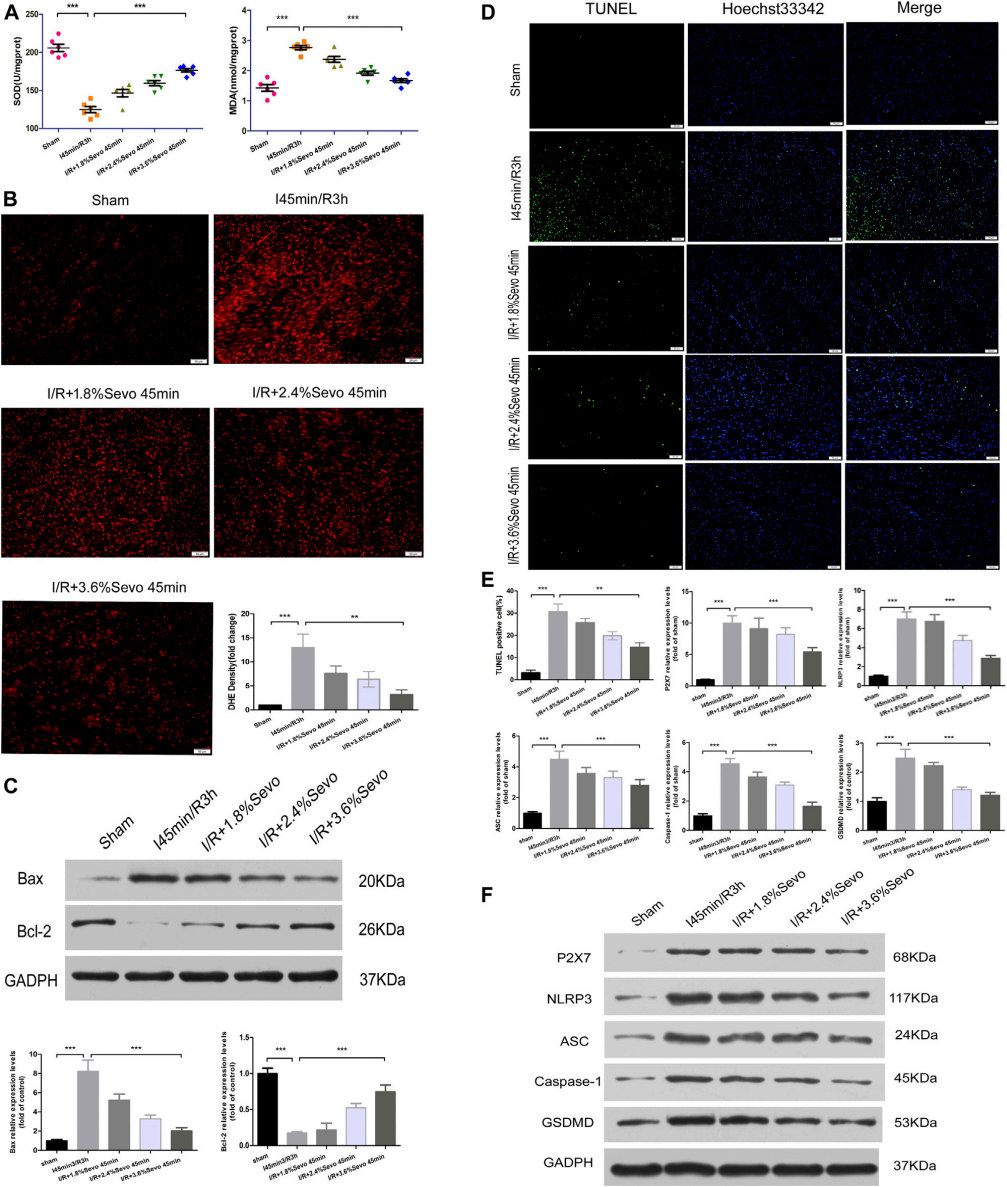
Sevoflurane alleviates oxidative stress and pyroptosis in MIRI rats **(A)** Effects of different concentrations of Sevoflurane on the expression of MDA and SOD in myocardial tissue after ischemia reperfusion. All values are expressed as means ± SD (n = 6). *p < 0.05, **p < 0.01, ***p < 0.001 vs respective controls **(B)** ROS Fluorescent Probe-DHE. Sevoflurane increased ATP content, stabilized membrane potential, and reduced ROS production (n = 6,Scale bars 50 um) **(C)** The Expression of apoptosis-related proteins Bax and Bcl-2.Data are expressed relative to the mean value for sham group and were presented as mean ± SD (n = 6). *p < 0.05, **p < 0.01, ***p < 0.001 vs respective controls **(D)** Effects of different concentrations of Sevoflurane on the TUNEL staining in myocardial tissue after MIRI. All values are expressed as means ± SD (n = 6). *p < 0.05, **p < 0.01, ***p < 0.001 vs respective controls. Scale bars 50 um **(E)** Histogram of TUNEL and the protein expression of P2X7, NLRP3, ASC, Caspase-1 and GSDMD **(F)** Immunoblotting measured the levels of P2X7, NLRP3, ASC, Caspase-1 and GSDMD protein expression. Data are expressed relative to the mean value for control group and were presented as mean ± SD (n = 6). *p < 0.05, **p < 0.01, ***p < 0.001 vs respective controls.

Superoxide dismutase (SOD) in myocardial tissue can transform reactive oxygen radicals into hydrogen peroxide, which is reduced to water and oxygen molecules by thixolase and glutathione peroxidase. SOD activity reflects the ability of scavenging reactive oxygen radicals. Methane dicarboxylic aldehyde (MDA) reflects the degree of lipid peroxidation on unsaturated fatty acids of cell membrane. Increased MDA can cause decreased membrane fluidity, increased permeability, mitochondrial swelling, lysosomal destruction and lysosomal enzyme release. The content of SOD and MDA indirectly reflects the ability of scavenging reactive oxyradical. Compared with the Sham group, SOD content in the I/R group decreased, and a large amount of Oxygen free radicals (OFR) produced during myocardial reperfusion could not be removed. Polyunsaturated fatty acids and unsaturated double bonds of fatty acids in the phospholipids of cell membranes were easily attacked by reactive oxygen radicals, leading to lipid peroxidation and stimulating the production of reactive oxygen radicals. In addition, the results showed that MDA levels increased, further aggravating the myocardial injury. However, in the Sevoflurane pretreatment group, SOD content increased, reducing lipid peroxidation of cell membrane structure, and reducing the amount of MDA. Through the increase of SOD and the elimination of MDA, myocardial cells can be protected from ischemia and reperfusion, and further damage of ischemic damaged cells can be alleviated during reperfusion (Figure 5A).

### DHE Staining of Rat Myocardial Tissue

Dihydroethidium (DHE) can enter living cells freely and is oxidized by intracellular Reactive oxygen species (ROS) to form ethidium oxide. The ethidium oxide can enter the chromosomal DNA and produce red fluorescence. The amount of ROS release was determined by observing the fluorescence intensity of DHE in frozen sections of myocardial tissue. Compared with the Sham group, the fluorescence intensity in the myocardial tissue of rats in the I/R group was significantly enhanced, the release of ROS was significantly increased, and the cardiac function was seriously damaged. Since the accumulation of ROS is a major factor that triggers the activation of NLRP3 inflammasomes, it can promote pro-caspase-1 cleavage and promote the release of inflammatory cytokines such as IL-1β and IL-18. Therefore, the intensity of ROS production is correlated with the activation of NLRP3 inflammasomes. Compared with the I/R group, the release degree of ROS in the myocardium of the Sevoflurane pretreated rats was reduced, indicating that Sevoflurane pretreatment can reduce the release of ROS and reduce the degree of oxidative stress (Figure 5B).

### Sevoflurane Alleviates Apoptosis of Cardiomyocytes

Apoptosis is an energy-dependent process and could be induced during ischemia which is affected by reperfusion (when oxygen supply is reinitiated) (Bellis et al., 2020). The apoptosis of cardiomyocytes is the main form of ischemia-reperfusion injury and the cytological basis for the occurrence and evolution of many cardiovascular diseases. The most important characteristic of apoptosis is that the plasma membrane of the cell remains intact throughout the whole process and there is no extracellular leakage of cell contents.Bcl-2 is located on the outer membrane of mitochondria and inhibits the release of cytochrome C, and its high expression can significantly inhibit apoptosis caused by the increase of free radicals, calcium overload and lipid peroxidation. Bax is a pro-apoptotic protein, located in the cytoplasm. After activation, it is translocated onto the mitochondrial membrane to form apoptotic protein complexes with Bcl-2. When Bax forms homodimer, cell apoptosis is induced, and when Bax and Bcl-2 form heterodimer, Bcl-2 inhibits cell apoptosis. Therefore, the balance of Bcl-2 and Bax is an important factor determining the degree of apoptosis in myocardial ischemia reperfusion injury. After myocardial ischemia and reperfusion, the expression of Bcl-2 was significantly decreased, the expression of Bax was significantly increased, and apoptosis was increased. The up-regulation of Bcl-2 or down-regulation of Bax can reduce cell apoptosis and prevent ischemia reperfusion injury. The results showed that pretreatment with Sevoflurane could increase the expression of Bcl-2, decrease the expression of Bax, increase the ratio of Bcl-2 to Bax, and reduce the apoptosis of cardiomyocytes (Figure 5C).

In apoptotic cells, DNA is broken down into fragments of different sizes. The TUNEL method connects both luciferin labeled and unlabeled DnTP to the fractured 3′-OH ends in apoptotic cells under the action of deoxyribonucleotide terminal transferase. Microscopically, specific green fluorescence could be detected in apoptotic cells. The results showed that there was little bright green fluorescence and almost no apoptosis in the Sham group. The green fluorescence of H/R group was significantly increased. Pretreatment with Sevoflurane significantly reduced the apoptosis of cardiomyocytes (Figure 5D).

### Sevoflurane Affects the Protein Expressions of P2X7, NLRP3, ASC, Caspase-1 and GSDMD

Pyroptosis is associated with inflammasome activation, production of caspase-1 and GSDMD, and release of a large number of pro-inflammatory cytokines. The protein expressions of P2X7, NLRP3, ASC, Caspase-1 and GSDMD were increased in myocardial tissue after MIRI. Different concentrations of Sevoflurane were used to observe the difference in protein expression of P2X7, NLRP3, ASC and Caspase-1. The results showed that the protein expression of P2X7, NLRP3, ASC and Caspase-1 was decreased with different concentrations of Sevoflurane, and related to the concentration of Sevoflurane. The expression of GSDMD protein was increased after MIRI, indicating the increase of pyroptosis and Sevoflurane reduced the degree of pyroptosis (Figure 5F).

## Sevoflurane Inhibited the Activation of Inflammasome and Pyroptosis Induced by Hypoxia and Reoxygenation of NRVM

### Sevoflurane Affects the mRNA Expression of P2X7 and NLRP3

**FIGURE 6:**
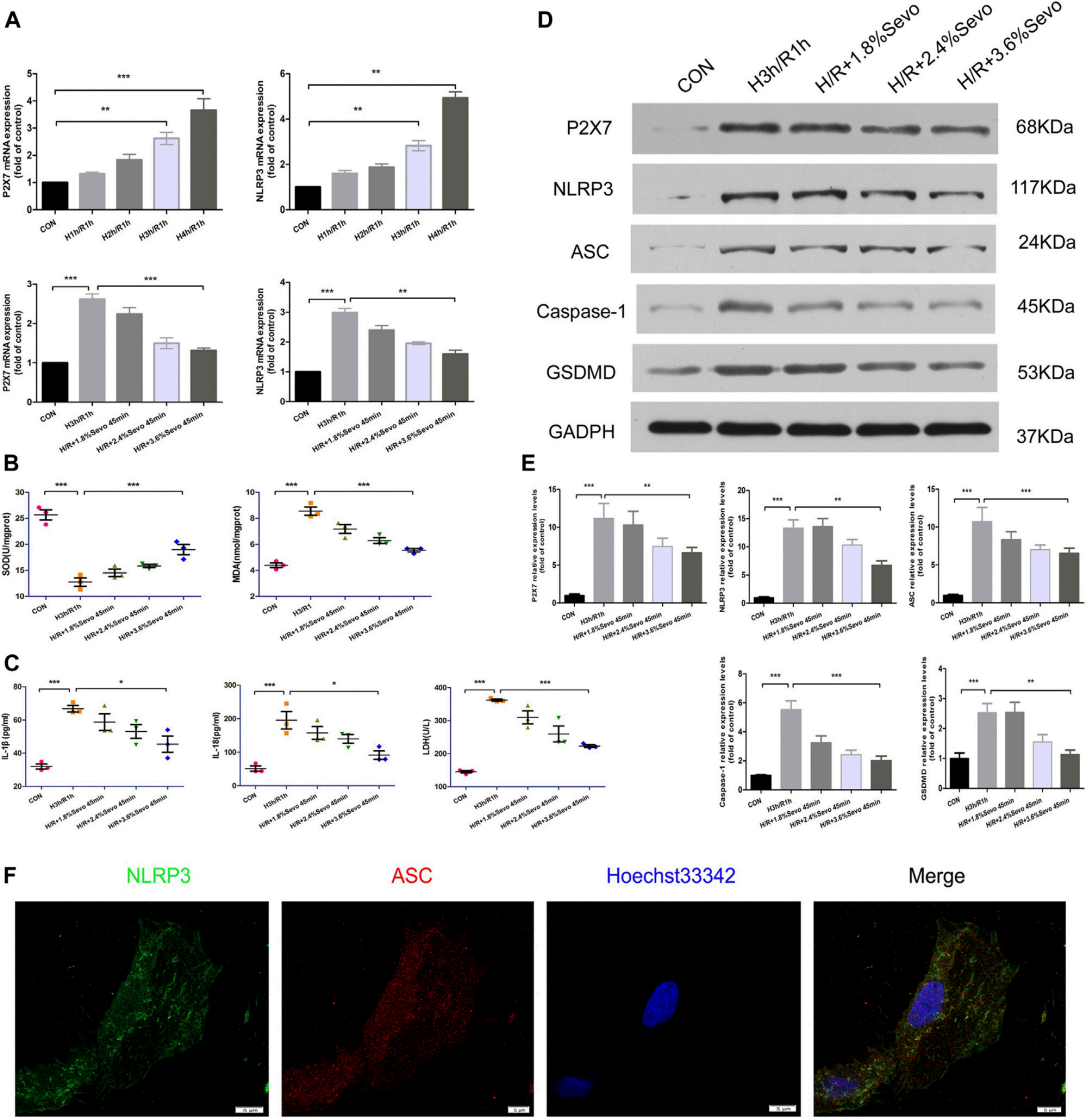
Sevoflurane inhibited the activation of inflammasome and pyroptosis induced by hypoxia and reoxygenation of NRVM **(A)** The expression of P2X7 and NLRP3 mRNA detected by real-time PCR. Data are expressed relative to the mean value for control group and were presented as mean ± SD (n = 3). *p < 0.05, **p < 0.01, ***p < 0.001 vs respective controls **(B)** Effects of different concentrations of Sevoflurane on the content of MDA and SOD. All values are expressed as mean ± SD (n = 3). *p < 0.05, **p < 0.01, ***p < 0.001 vs respective controls **(C)** Effects of Sevoflurane at different concentrations on the release of IL-1β, IL-18 and LDH in NRVM after H/R. All values are expressed as mean ± SD (n = 3). *p < 0.05, **p < 0.01, ***p < 0.001 vs respective controls **(D)** Effects of Sevoflurane at different concentrations on the protein expression of P2X7,NLRP3,ASC,Caspase-1 and GSDMD in NRVM after H/R by immunoblotting analysis **(E)** Histogram of the protein expression of P2X7,NLRP3,ASC,Caspase-1 and GSDMD. Data are expressed relative to the mean value for control group and were presented as mean ± SD (n = 3). *p < 0.05, **p < 0.01, ***p < 0.001 vs respective controls **(F)** Colocalization of NLRP3 and ASC in NRVM was observed by the laser scanning confocal microscope(n = 3,Scale bars five um).

The expression of P2X7 and NLRP3 mRNA increased after H/R in NRVM, and the expression of NLRP3 mRNA increased gradually with the prolongation of hypoxia time. In order to confirm the effect of Sevoflurane on the gene expression of NLRP3, we examined the effect of Sevoflurane at different concentrations on the mRNA expression of P2X7 and NLRP3. The results showed that Sevoflurane treatment inhibited the mRNA expression of P2X7 and NLRP3, and the degree of inhibition was related to the concentration of Sevoflurane (Figure 6A).

### Sevoflurane Alleviates the Release of Cell Inflammatory Factors and Myocardial Enzyme

The levels of MDA and SOD after H/R were determined by ELISA. After H/R of NRVM, the levels of MDA in the supernatant of the culture medium were significantly increased, and the level of SOD decreased. Compared with H/R group, Sevoflurane with different concentrations reduced the content of MDA in NRVM and increased the content of SOD. 3.6%Sevoflurane group had the most protective effect on H/R (Figure 6B).

### Sevoflurane Alleviates the Release of Cell Inflammatory Factors and Myocardial Enzyme

The levels of IL-1β and IL-18 secretion and the release of LDH after cell injury were determined by ELISA. After hypoxia and reoxygenation of NRVM, the levels of IL-1β and IL-18 in the supernatant of the culture medium were significantly increased. At the same time, the release of LDH also increased. Compared with H/R group, Sevoflurane with different concentrations inhibited the secretion of IL-1β and IL-18, and reduced the release of LDH, and 3.6%Sevoflurane had the most obvious protective effect on MIRI (Figure 6C and Table 5).

**TABLE 5:** Effects of Sevoflurane at different concentrations on the release of IL-1β, IL-18 and LDH in NRVM after H/R (n = 3).

	IL-1β(pg/ml)	IL-18 (pg/ml)	LDH(U/L)
CON	30.98 ± 1.89^#^	51.29 ± 11.16^#^	145.25 ± 5.03^#^
H3h/R1h	66.74 ± 2.71^*^	195.41 ± 37.03^*^	362.39 ± 4.63^*^
H/R+1.8%Sevo 45 min	58.62 ± 7.11^*#^	157.79 ± 27.01^*#^	309.99 ± 27.58^*#^
H/R+2.4%Sevo 45 min	53.11 ± 5.96^*#^	139.78 ± 18.81^*#^	259.92 ± 34.38^*#^
H/R+3.6%Sevo 45 min	45.49 ± 6.91^*#^	91.63 ± 18.02^*#^	223.36 ± 5.53^*#^

*p < 0.05 vs CON group; #*p* < 0.05 vs H3h/R1h group.

### Sevoflurane Affects the Protein Expressions of P2X7, NLRP3, ASC, Caspase-1 and GSDMD in NRVM

The protein expressions of P2X7, NLRP3, ASC, Caspase-1 and GSDMD were increased in the NRVM after H/R. Different concentrations of Sevoflurane were used to observe the difference in protein expression level of P2X7, NLRP3, ASC and Caspase-1. The results showed that the protein expressions of P2X7, NLRP3, ASC and Caspase-1 were decreased with different concentrations of Sevoflurane. The expression of GSDMD protein was increased after H/R, indicating the increase of pyroptosis, and Sevoflurane treatment reduced the degree of pyroptosis (Figures 6D, E).

### Colocalization of NLRP3 and ASC in NRVM

In the absence of stimuli, intracellular caspase-1 exists in the form of inactive pro-caspase-L. In the presence of pathological factors, NLRP3 recruits effector protein Pro-Caspase-1 through ASC to assemble into inflammasomes. After assembly, inflammasomes can be self-activated to hydrolyze Pro-Caspase-1 into active Caspase-L. In the immunofluorescence experiment of cells, confocal microscopy was used for observation. After hypoxia and reoxygenation of NRVM, it was observed that NLRP3 and ASC were co-located in the cytoplasm, and formed spots (Figure 6F).

## Effects of NLRP3 Inhibitor and Agonist in NRVM After Sevoflurane Pretreatment

### Changes in Protein Expression of NLRP3, ASC, Caspase-1 and GSDMD in NRVM

**FIGURE 7:**
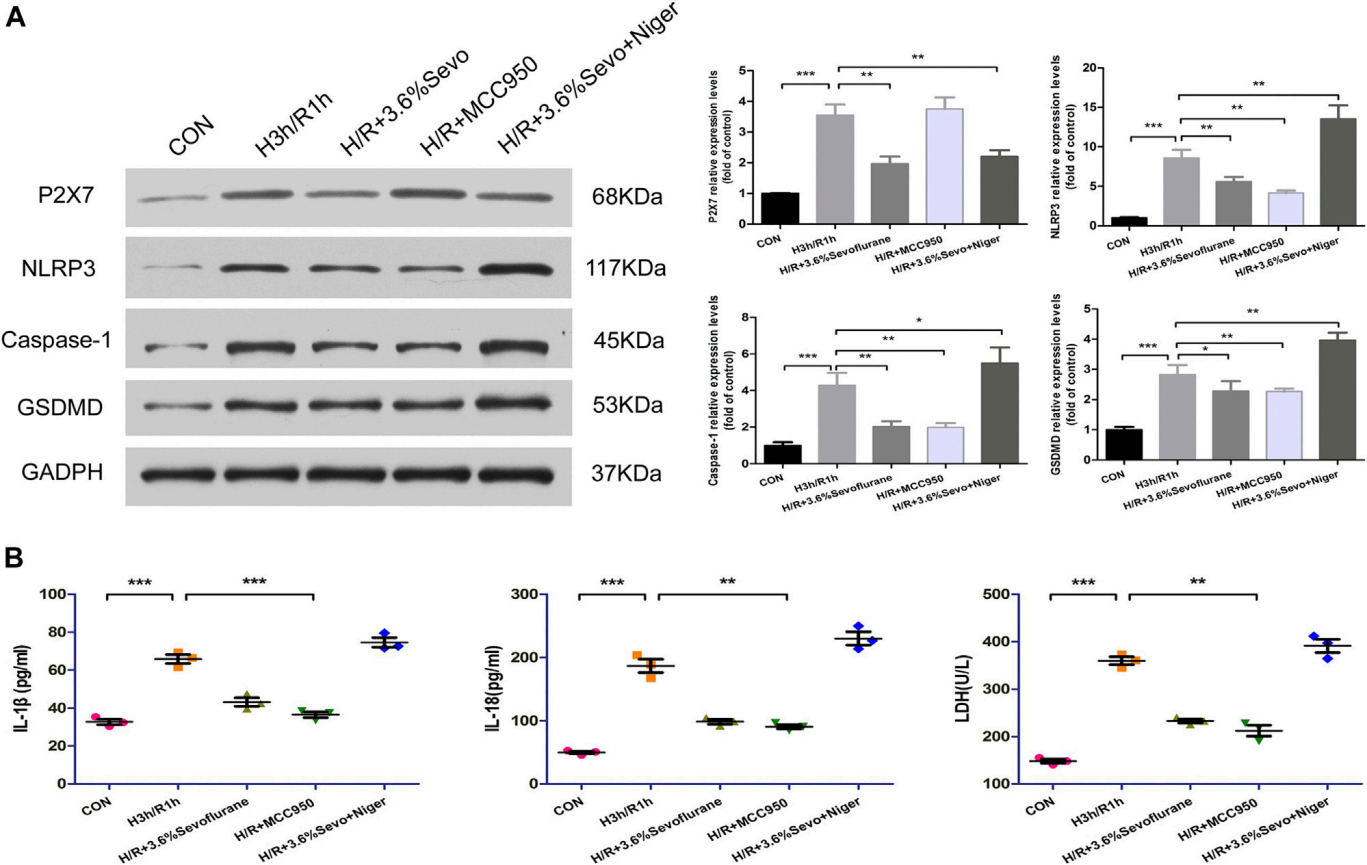
Effects of NLRP3 inhibitor and agonist on NRVM after Sevoflurane pretreatment **(A)** Effects of NLRP3 inhibitor and agonist on the protein expression of P2X7, NLRP3, Caspase-1 and GSDMD in NRVM after Sevoflurane pretreatment by immunoblotting. Data are expressed relative to the mean value for control group and were presented as mean ± SD (n = 3). *p < 0.05, **p < 0.01, ***p < 0.001 vs respective controls **(B)** Effects of NLRP3 agonists and inhibitors on the levels of IL-1β, IL-18 and LDH after H/R of cardiomyocytes. All values are expressed as mean ± SD (n = 3). *p < 0.05, **p < 0.01, ***p < 0.001 vs respective controls.

After hypoxia and reoxygenation, the protein expressions of P2X7, NLRP3, Caspase-1 and GSDMD were increased. MCC950, an inhibitor of NLRP3, inhibited the up-regulation of NLRP3, Caspase-1 and GSDMD induced by H/R.As an agonist of NLRP3, Nigericin affected the inhibitory effect of Sevoflurane on the protein expression of P2X7, NLRP3, caspase-1 and GSDMD, and up-regulated the expressions of NLRP3, caspase-1 and GSDMD (Figure 7A).

### The Release of Inflammatory Factors and Myocardial Enzyme

After hypoxia and reoxygenation, the levels of IL-1β, IL-18 and LDH in the supernatant of the culture medium were significantly increased, while the secretion was inhibited by Sevoflurane treatment. In order to determine the effect of Sevoflurane on cardiomyocyte hypoxia and reoxygenation injury, we used NLRP3 inhibitors and agonists on cardiomyocytes after hypoxia and reoxygenation, and observed the changes of NLRP3 related factors. The results showed that the use of NLRP3 inhibitor MCC950 100 nM, can inhibit the secretion of inflammatory factors IL-1β and IL-18, reduce the release of LDH; NLRP3 agonist Nigericin 10 uM increased the secretion of IL-1β, IL-18 and LDH (Figure 7B).

## Discussion

This study included three aspects:1) the important role of cardiomyocyte pyrolysis in MIRI, 2) the effect of Sevoflurane on MIRI from both *in vivo* and *in vitro* models. And 3) Inhibition of cardiomyocyte inflammation and pyroptosis via the P2X7-NLRP3 signaling pathway is the potential mechanism of Sevoflurane against MIRI. These results suggest that Sevoflurane has potential clinical value in the prevention and reversal of MIRI.

The mechanism of MIRI was extensively explored. The role of inflammatory factors in ischemia-reperfusion injury has been widely studied (Toldo et al., 2018). Most animal experiments have shown that the expression of proinflammatory factors increases during myocardial ischemia-reperfusion (Silvis et al., 2021).IL-1β can cooperate with other cytokines to promote the activation of B and T cells, and induce the production of other inflammatory mediators, strengthen the adhesion of white blood cells and endothelial cells. MIRI regulates the generation of IL-6 and NF-kB (Fiordelisi et al., 2019), and activates the cytokine cascade reaction (Jia et al., 2020). It can promote the generation of oxygen free radicals (Hu et al., 2008), activate neutrophils, and induce the apoptosis of cardiomyocytes (Lopez-Neblina and Toleto-Pereyra, 2007; Jun et al., 2019). Although myocardial inflammation occurs after ischemia/reperfusion and leads to myocardial cell damage, the cellular/molecular and signaling mechanisms of inflammation remain unclear. Almost all of the factors that induce inflammasome may be associated with MIRI, including reactive oxygen species (ROS) (Abderrazak et al., 2015; Zhang et al., 2018), oxygen-low-density lipoprotein, hypoxia, complement, amyloid and misfolded proteins, especially in macrophages and immune cells. In our findings, we found that inflammation and pyrolysis of cardiomyocytes after MIRI may be factors that accelerate the progression of myocardial ischemia/reperfusion injury. We propose a hypothesis that cardiomyocytes produce pyrolysis after myocardial ischemia reperfusion, and Sevoflurane reduces cellular inflammation and pyrolysis by inhibiting NLRP3 expression, which explains the anti-MIRI effect of Sevoflurane.

Sevoflurane is a commonly used inhaled anesthetic in clinical practice. Compared with other inhaled anesthetics, Sevoflurane has less effect on the hemodynamics and is easy to adjust the depth of anesthesia. Therefore, Sevoflurane is widely used in anesthesia induction and maintenance. In clinical practice, Sevoflurane has been found to have an protective effect on myocardial ischemia injury (Grishin et al., 2016; Zhang et al., 2017; Zhang et al., 2020), and this finding was supported by relevant studies. However, the mechanism of Sevoflurane against myocardial ischemia has not been determined. By measuring the changes of injury factors in serum of patients pre-anesthesia and post-anesthesia, we found that the effect of Sevoflurane on myocardial ischemia injury was related to anti-inflammation. In order to investigate the anti-inflammatory mechanism of Sevoflurane, we found that NLRP3, ASC, cleaved caspase-1, and GSDMD N-terminal fragments were significantly upregulated in cardiomyocytes in animal and cell experiments (Sandanger et al., 2016). In addition, the release of IL-1β and IL-18 from cardiomyocytes was increased. Sevoflurane can inhibit the overexpression of P2X7, NLRP3, ASC, Caspase-1 and GSDMD in animal MIRI and primary cardiomyocyte H/R models, reduce the inflammatory response and cell pyroptosis induced by H/R, and improve the function of cardiomyocytes.

In addition, when Sevoflurane is pretreated on cardiomyocytes, the concentration of inhalation, the time of administration and the duration of action all have important effects on its myocardial protective effect (Miyamae et al., 2009). According to the study of Miyamae, after Sevoflurane pretreatment, the elution time should not exceed 60min, otherwise the myocardial protective effect will be lost. This suggests that appropriate Sevoflurane treatment is the key to the myocardial protective effect (Obal et al., 1987). According to the results of previous studies, the concentration of Sevoflurane pretreatment before ischemia or hypoxia was selected to be 3.6% and the action time was 45 min for the release of inflammasomes and downstream factors. Restraining the degree of burnout. Pyroptosis has the characteristics of necrosis and apoptosis in morphology, but different from apoptosis is the formation of many pores on the cell membrane, so that the cell membrane lost its integrity (Willingham et al., 2009). The GSDMD molecule is the main agent of cell pyrolysis. Caspase-1 cleaves GSDMD (GSDMD-FL) to produce the GSDMD-N terminal, which inserts into the cell membrane to form a 10–14 nm pore.Caspase-1 then further cleaves Pro-IL-1β and Pro-IL-18, causing the maturation and secretion of IL-1β and IL-18, amplifying local and systemic inflammatory responses, and ultimately leading to cell death (Mallat et al., 2001; Wu et al., 2018). Activation of inflammasome and its downstream inflammatory signaling molecules is an important pathway to induce myocardial inflammation after ischemia (Baldrighi et al., 2017). Both the occurrence of pyroptosis and the release of IL-1β and IL-18 will aggravate MIRI.

In summary, our study showed that the protective effect of Sevoflurane against myocardial ischemia injury is associated with anti-inflammation. The effect of Sevoflurane was associated with inhibition of inflammasome, IL-1β, IL-18 and cell pyroptosis.

## Data Availability

The raw data supporting the conclusions of this article will be made available by the authors, without undue reservation.
