# Corrigendum: Sevoflurane Alleviates Myocardial Ischemia Reperfusion Injury by Inhibiting P2X7-NLRP3 Mediated Pyroptosis

**DOI:** 10.3389/fmolb.2022.901322

**Published:** 2022-04-13

**Authors:** Jiaxuan Wu, Wenfeng Cai, Ruiming Du, Haiyang Li, Bin Wang, Yanqiong Zhou, Daifei Shen, Huimin Shen, Yang Lan, Lesi Chen, Xiaoxia Zheng, Danmei Huang, Ganggang Shi

**Affiliations:** ^1^ Department of Anesthesiology, Second Affiliated Hospital of Shantou University Medical College, Shantou, China; ^2^ Department of Pharmacology, Shantou University Medical College, Shantou, China

**Keywords:** sevoflurane, myocardial ischemia reperfusion, hypoxia and reoxygenation, P2X7, NLRP3, pyroptosis

There is an error in the **Funding** statement. The correct number for “the National Natural Science Foundation of China” is “ 81473215 and 81870276”.

Additionally, in the original article, there was a mistake in “Figures 2–4” as published. The problem was caused by the incomplete uploading of some figures in the process of uploading revised manuscripts and figures before publication. We did not carefully check it, which led to the error when publishing the article. The correct figures appear below:

The authors apologize for this error and state that this does not change the scientific conclusions of the article in any way. The original article has been updated.

**FIGURE 2:**
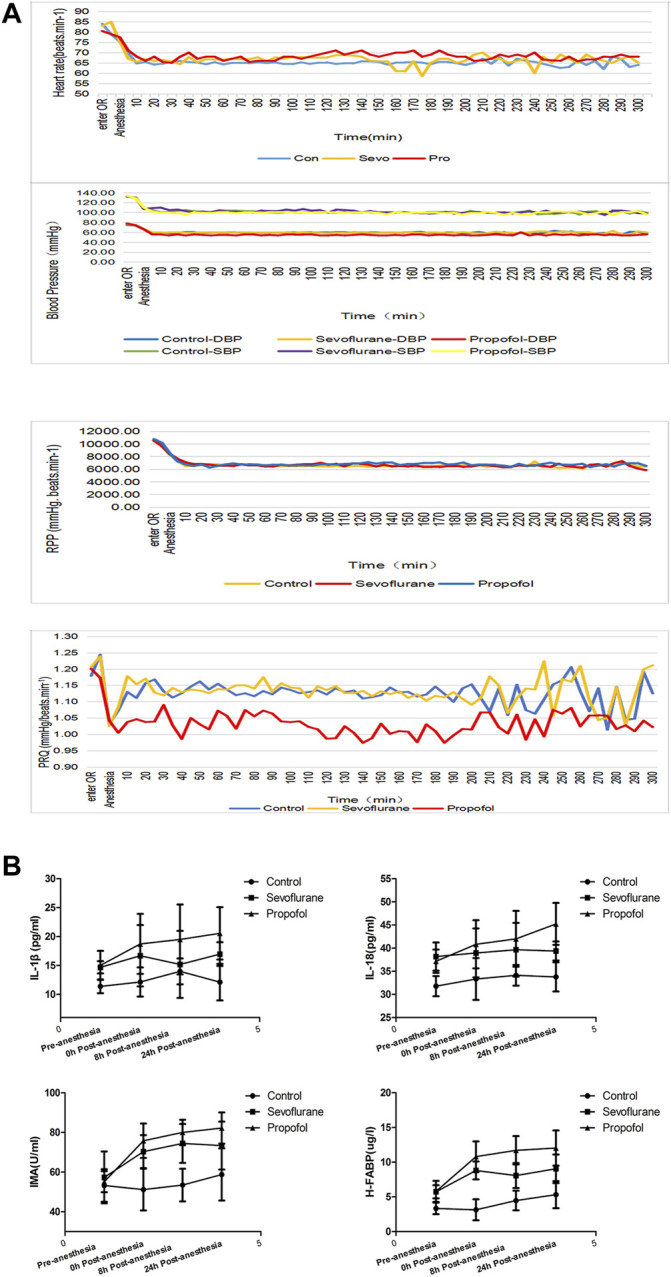
Changes in vital signs and inflammatory factors in these three groups of patients**.** **(A)** different general anesthetics on the changes of heart rate, blood pressure, RPP and PRQ in peri-anesthesia in the three groups. **(B)** Changes of IL-18 at different time points at pre- and post-anesthesia.

**FIGURE 3:**
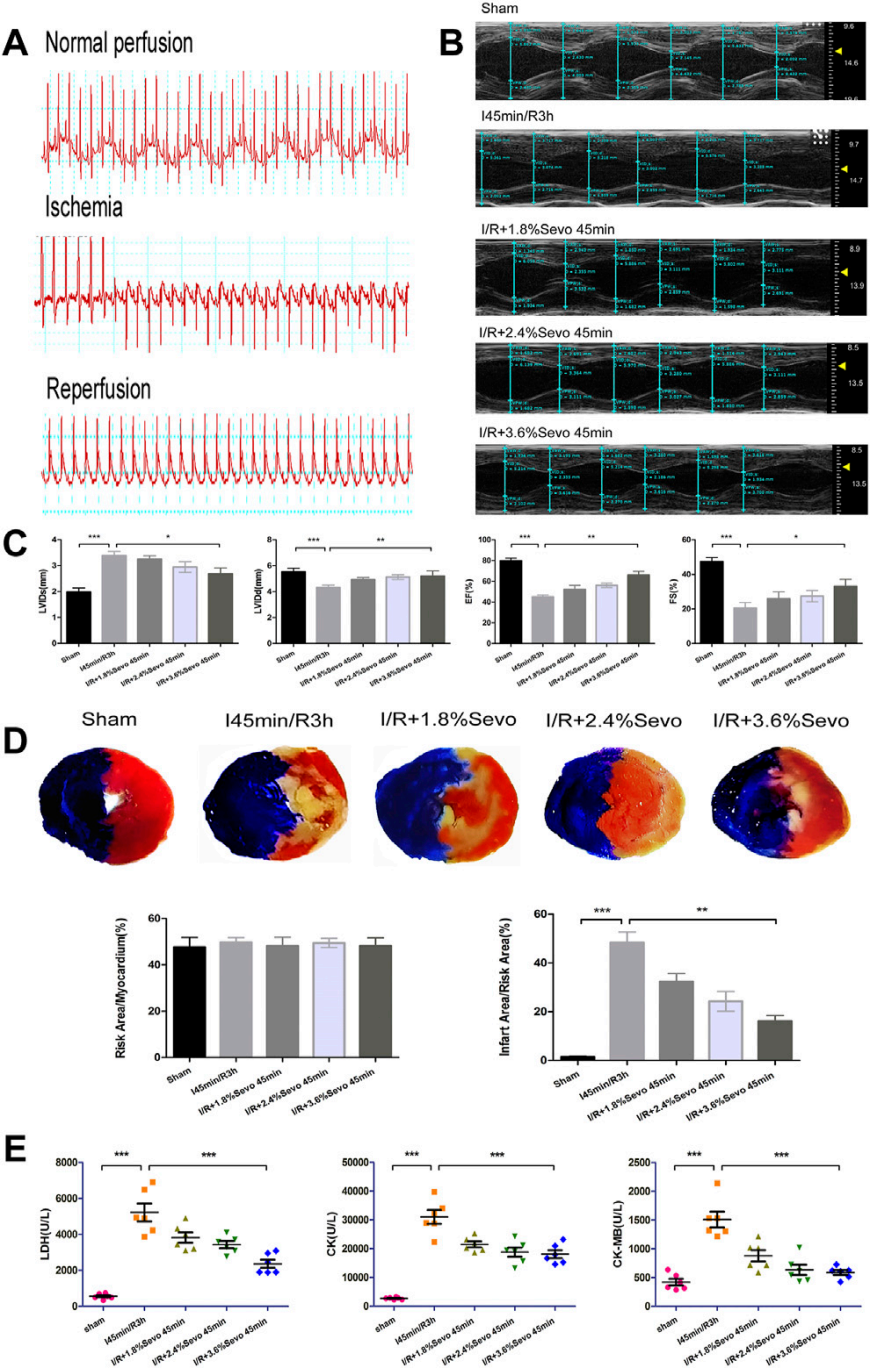
Sevoflurane alleviates MIRI in rats. **(A)** Typical electrocardiogram of normal perfusion, ligation of LAD and reperfusion. **(B)** Cardiac ultrasonography in rats of normal perfusion, MIRI and treatment with different concentrations of sevoflurane. **(C)** Histogram of rat cardiac ultrasonography. **(D)** Myocardial infarction area and histogram of rats treated with different concentrations of sevoflurane. **(E)** Effects of Sevoflurane at different concentrations on the release of LDH, CK, and CK-MB in myocardial tissue after MIRI. Data are expressed relative to the mean value of sham group and were presented as mean ± SD (n = 6). *p < 0.05, **p < 0.01, ***p < 0.001 vs. respective controls.

**FIGURE 4:**
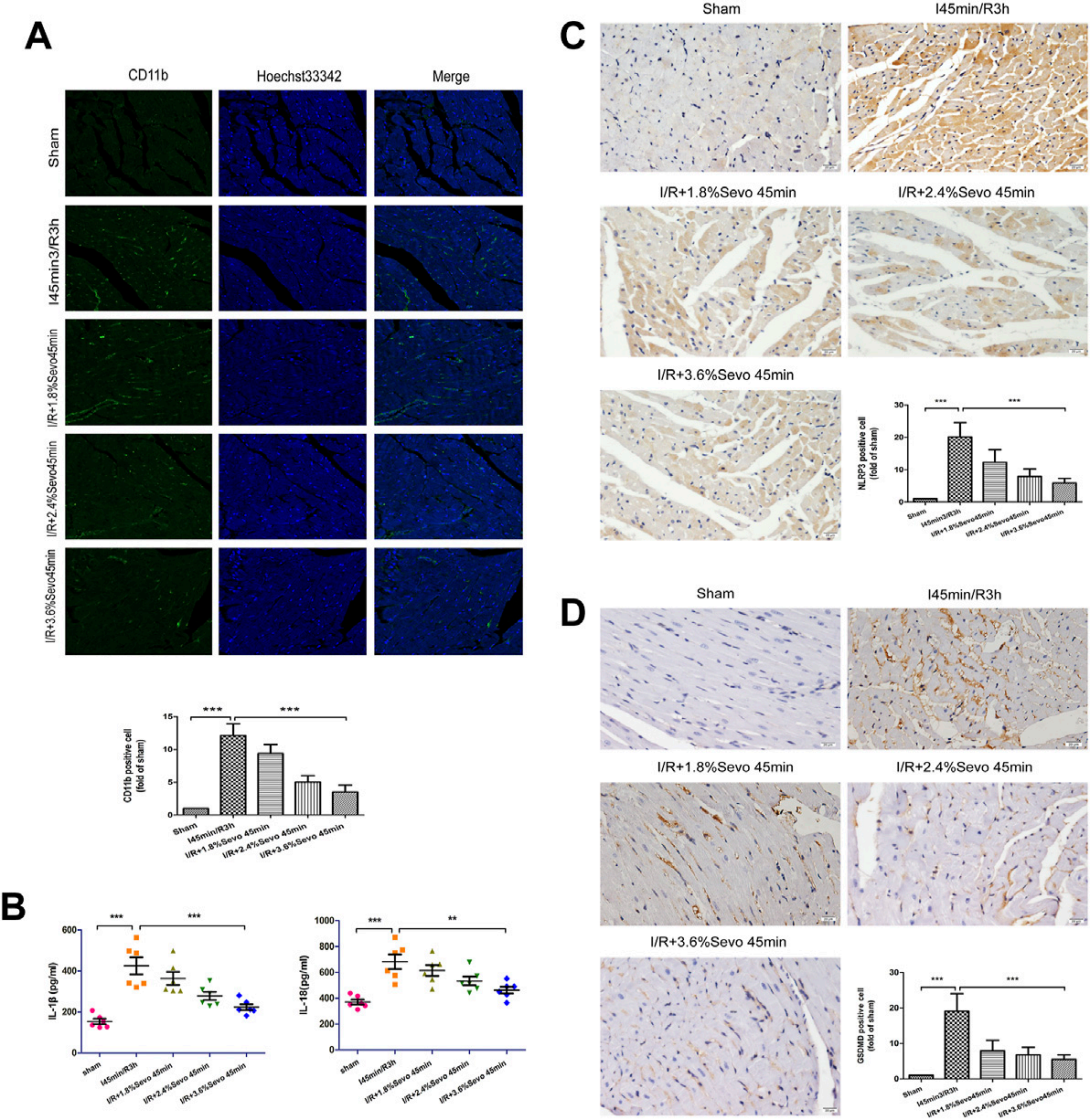
Sevoflurane alleviates inflammatory cell infiltration in MIRI rats. **(A)** The expression of CD11b in rat myocardial tissue determined by immunofluorescence staining (n = 6, Scale bars 20 µm). **(B)** Effects of Sevoflurane at different concentrations on the release of IL-1β and IL-18 in myocardial tissue after MIRI. All values are expressed as means ± SD (n = 6). *p < 0.05, **p < 0.01, ***p < 0.001 vs. respective controls. **(C)** Expression of NLRP3 in rat myocardial tissue by immunohistochemical staining of normal perfusion, MIRI and sevoflurane treatment with different concentrations in rats (n = 6, Scale bars 20 µm). **(D)** The Expression of GSDMD in rat myocardial tissue by immunohistochemical staining of normal perfusion, MIRI and sevoflurane treatment with different concentrations in rats (n = 6, Scale bars 20 µm). Data are expressed relative to the mean value for sham group and were presented as mean ± SD. *p < 0.05, **p < 0.01, ***p < 0.001 vs. respective controls.

